# Supplementary material for: Modeling glioblastoma heterogeneity as a dynamic network of cell states
Source: Mol Syst Biol. 2021 Sep 16;17(9):e10105. doi: 10.15252/msb.202010105 (PMC8444284; doi:10.15252/msb.202010105)
Supplement: Supplementary file 6 — Source Data for Figure 5 [file MSB-17-e10105-s004.zip › Figure5A_sourcedata/GSEA_3071/hallmarks_stateV.GseaPreranked.1623742546329/HALLMARK_MYC_TARGETS_V1.html]

Details for gene set HALLMARK\_MYC\_TARGETS\_V1[GSEA]

|  || Dataset | state43071 |
| Phenotype | NoPhenotypeAvailable |
| Upregulated in class | na\_pos |
| GeneSet | HALLMARK\_MYC\_TARGETS\_V1 |
| Enrichment Score (ES) | 0.2543459 |
| Normalized Enrichment Score (NES) | 1.3887718 |
| Nominal p-value | 0.05733558 |
| FDR q-value | 0.08616507 |
| FWER p-Value | 0.882 |
Table: GSEA Results Summary

  

Fig 1: Enrichment plot: HALLMARK\_MYC\_TARGETS\_V1      
 Profile of the Running ES Score & Positions of GeneSet Members on the Rank Ordered List

  

| PROBE | GENE SYMBOL | GENE\_TITLE | RANK IN GENE LIST | RANK METRIC SCORE | RUNNING ES | CORE ENRICHMENT || 1 | PCNA |  |  | 10 | 1.908 | 0.0278 | Yes |
| 2 | MCM4 |  |  | 17 | 1.815 | 0.0549 | Yes |
| 3 | TYMS |  |  | 30 | 1.683 | 0.0788 | Yes |
| 4 | MCM7 |  |  | 88 | 1.400 | 0.0896 | Yes |
| 5 | DEK |  |  | 94 | 1.384 | 0.1102 | Yes |
| 6 | RRM1 |  |  | 99 | 1.364 | 0.1307 | Yes |
| 7 | RFC4 |  |  | 171 | 1.227 | 0.1361 | Yes |
| 8 | USP1 |  |  | 202 | 1.168 | 0.1485 | Yes |
| 9 | CDC45 |  |  | 228 | 1.138 | 0.1614 | Yes |
| 10 | CDK2 |  |  | 233 | 1.134 | 0.1783 | Yes |
| 11 | MCM2 |  |  | 238 | 1.130 | 0.1951 | Yes |
| 12 | MCM5 |  |  | 243 | 1.127 | 0.2119 | Yes |
| 13 | MCM6 |  |  | 282 | 1.080 | 0.2214 | Yes |
| 14 | CCNA2 |  |  | 309 | 1.057 | 0.2329 | Yes |
| 15 | DUT |  |  | 314 | 1.050 | 0.2484 | Yes |
| 16 | MAD2L1 |  |  | 421 | 0.932 | 0.2425 | Yes |
| 17 | KPNA2 |  |  | 435 | 0.919 | 0.2543 | Yes |
| 18 | TRA2B |  |  | 596 | 0.802 | 0.2360 | No |
| 19 | HNRNPD |  |  | 659 | 0.769 | 0.2360 | No |
| 20 | TFDP1 |  |  | 711 | 0.742 | 0.2377 | No |
| 21 | SRSF7 |  |  | 921 | 0.650 | 0.2076 | No |
| 22 | HNRNPA3 |  |  | 954 | 0.638 | 0.2114 | No |
| 23 | RANBP1 |  |  | 999 | 0.620 | 0.2125 | No |
| 24 | RNPS1 |  |  | 1035 | 0.612 | 0.2153 | No |
| 25 | SMARCC1 |  |  | 1049 | 0.608 | 0.2223 | No |
| 26 | SRSF2 |  |  | 1084 | 0.597 | 0.2250 | No |
| 27 | CNBP |  |  | 1370 | 0.514 | 0.1781 | No |
| 28 | HNRNPU |  |  | 1548 | 0.470 | 0.1513 | No |
| 29 | UBA2 |  |  | 1612 | 0.457 | 0.1463 | No |
| 30 | PRPS2 |  |  | 1702 | 0.435 | 0.1359 | No |
| 31 | SNRPG |  |  | 1825 | 0.414 | 0.1188 | No |
| 32 | PPM1G |  |  | 2017 | 0.385 | 0.0880 | No |
| 33 | XPO1 |  |  | 2091 | 0.374 | 0.0798 | No |
| 34 | PA2G4 |  |  | 2112 | 0.372 | 0.0817 | No |
| 35 | HNRNPR |  |  | 2175 | 0.365 | 0.0754 | No |
| 36 | NCBP1 |  |  | 2271 | 0.354 | 0.0626 | No |
| 37 | HPRT1 |  |  | 2404 | 0.334 | 0.0424 | No |
| 38 | SRSF1 |  |  | 2505 | 0.322 | 0.0281 | No |
| 39 | HNRNPA2B1 |  |  | 2523 | 0.320 | 0.0298 | No |
| 40 | SF3B3 |  |  | 2649 | 0.305 | 0.0105 | No |
| 41 | LSM7 |  |  | 2653 | 0.304 | 0.0146 | No |
| 42 | SRSF3 |  |  | 2732 | 0.297 | 0.0042 | No |
| 43 | SNRPA |  |  | 2738 | 0.296 | 0.0079 | No |
| 44 | VDAC3 |  |  | 2803 | 0.289 | 0.0000 | No |
| 45 | BUB3 |  |  | 2806 | 0.288 | 0.0041 | No |
| 46 | CBX3 |  |  | 2851 | 0.284 | 0.0001 | No |
| 47 | NDUFAB1 |  |  | 3018 | 0.267 | -0.0278 | No |
| 48 | DHX15 |  |  | 3022 | 0.267 | -0.0242 | No |
| 49 | EIF4G2 |  |  | 3254 | -0.260 | -0.0647 | No |
| 50 | PSMD3 |  |  | 3261 | -0.261 | -0.0618 | No |
| 51 | PGK1 |  |  | 3286 | -0.263 | -0.0623 | No |
| 52 | SNRPB2 |  |  | 3289 | -0.264 | -0.0586 | No |
| 53 | PSMC6 |  |  | 3338 | -0.274 | -0.0636 | No |
| 54 | PHB |  |  | 3365 | -0.277 | -0.0643 | No |
| 55 | PSMD7 |  |  | 3391 | -0.280 | -0.0647 | No |
| 56 | PSMB3 |  |  | 3413 | -0.284 | -0.0644 | No |
| 57 | CCT2 |  |  | 3510 | -0.301 | -0.0782 | No |
| 58 | EEF1B2 |  |  | 3605 | -0.316 | -0.0914 | No |
| 59 | PSMC4 |  |  | 3608 | -0.317 | -0.0868 | No |
| 60 | NME1 |  |  | 3615 | -0.317 | -0.0830 | No |
| 61 | MRPL9 |  |  | 3626 | -0.319 | -0.0800 | No |
| 62 | C1QBP |  |  | 3672 | -0.326 | -0.0836 | No |
| 63 | PSMA7 |  |  | 3678 | -0.327 | -0.0795 | No |
| 64 | MRPS18B |  |  | 3719 | -0.334 | -0.0820 | No |
| 65 | AP3S1 |  |  | 3729 | -0.336 | -0.0785 | No |
| 66 | SLC25A3 |  |  | 3743 | -0.338 | -0.0757 | No |
| 67 | CAD |  |  | 3792 | -0.348 | -0.0795 | No |
| 68 | EIF2S1 |  |  | 3809 | -0.351 | -0.0772 | No |
| 69 | PABPC1 |  |  | 3893 | -0.366 | -0.0875 | No |
| 70 | RSL1D1 |  |  | 3915 | -0.369 | -0.0858 | No |
| 71 | CCT7 |  |  | 3954 | -0.377 | -0.0872 | No |
| 72 | CCT5 |  |  | 3958 | -0.379 | -0.0819 | No |
| 73 | UBE2L3 |  |  | 3977 | -0.384 | -0.0794 | No |
| 74 | RACK1 |  |  | 4022 | -0.393 | -0.0817 | No |
| 75 | SERBP1 |  |  | 4059 | -0.401 | -0.0824 | No |
| 76 | ETF1 |  |  | 4072 | -0.403 | -0.0785 | No |
| 77 | LDHA |  |  | 4145 | -0.421 | -0.0858 | No |
| 78 | CCT3 |  |  | 4162 | -0.427 | -0.0822 | No |
| 79 | EIF4A1 |  |  | 4210 | -0.442 | -0.0844 | No |
| 80 | ABCE1 |  |  | 4239 | -0.452 | -0.0828 | No |
| 81 | AIMP2 |  |  | 4252 | -0.457 | -0.0779 | No |
| 82 | EIF3B |  |  | 4253 | -0.457 | -0.0708 | No |
| 83 | RPS10 |  |  | 4340 | -0.485 | -0.0799 | No |
| 84 | SSB |  |  | 4400 | -0.501 | -0.0834 | No |
| 85 | CCT4 |  |  | 4407 | -0.503 | -0.0767 | No |
| 86 | PSMA1 |  |  | 4433 | -0.515 | -0.0735 | No |
| 87 | RPS5 |  |  | 4475 | -0.529 | -0.0732 | No |
| 88 | RPLP0 |  |  | 4483 | -0.532 | -0.0663 | No |
| 89 | NOP16 |  |  | 4494 | -0.539 | -0.0598 | No |
| 90 | RPL18 |  |  | 4532 | -0.555 | -0.0583 | No |
| 91 | SSBP1 |  |  | 4543 | -0.561 | -0.0515 | No |
| 92 | HSP90AB1 |  |  | 4563 | -0.570 | -0.0463 | No |
| 93 | PSMB2 |  |  | 4594 | -0.583 | -0.0430 | No |
| 94 | XPOT |  |  | 4596 | -0.586 | -0.0340 | No |
| 95 | RPS3 |  |  | 4632 | -0.610 | -0.0313 | No |
| 96 | PWP1 |  |  | 4635 | -0.611 | -0.0221 | No |
| 97 | RPL6 |  |  | 4649 | -0.618 | -0.0150 | No |
| 98 | SRM |  |  | 4653 | -0.620 | -0.0060 | No |
| 99 | RPL34 |  |  | 4660 | -0.624 | 0.0026 | No |
| 100 | EIF3D |  |  | 4676 | -0.631 | 0.0095 | No |
| 101 | EIF2S2 |  |  | 4683 | -0.635 | 0.0183 | No |
| 102 | EIF3J |  |  | 4741 | -0.671 | 0.0177 | No |
| 103 | RPS6 |  |  | 4751 | -0.678 | 0.0266 | No |
| 104 | PABPC4 |  |  | 4816 | -0.728 | 0.0256 | No |
| 105 | PRDX4 |  |  | 4821 | -0.732 | 0.0362 | No |
| 106 | RPL22 |  |  | 4838 | -0.747 | 0.0448 | No |
| 107 | DDX21 |  |  | 4853 | -0.757 | 0.0538 | No |
| 108 | MYC |  |  | 5020 | -0.972 | 0.0370 | No |
| 109 | NPM1 |  |  | 5040 | -1.017 | 0.0492 | No |
Table: GSEA details [plain text format]

  

Fig 2: HALLMARK\_MYC\_TARGETS\_V1: Random ES distribution      
 Gene set null distribution of ES for **HALLMARK\_MYC\_TARGETS\_V1**
